# *Let-7d* and *miR-185* Impede Epithelial-Mesenchymal Transition by Downregulating *Rab25* in Breast Cancer

**DOI:** 10.31557/APJCP.2021.22.1.305

**Published:** 2021-01

**Authors:** Arman Shahabi, Behrooz Naghili, Khalil Ansarin, Maryam Montazeri, Mehdi Dadashpour, Nosratollah Zarghami

**Affiliations:** 1 *Infectious and Tropical Diseases Research Center, Tabriz University of Medical Sciences, Tabriz, Iran. *; 2 *Department of Molecular Medicine, Faculty of Advanced Medical Sciences, Tabriz University of Medical Sciences, Tabriz, Iran. *; 3 *Tuberculosis and Lung Diseases Research Center, Tabriz University of Medical Sciences, Tabriz, Iran. *; 4 *Department of Medical Biotechnology, Faculty of Advanced Sciences and Technology, Tehran Medical Sciences, Islamic Azad University, Tehran, Iran. *; 5 *Stem Cell Research Center, Tabriz University of Medical Sciences, Tabriz, Iran. *

**Keywords:** Breast cancer, epithelial, mesenchymal transition, Let-7d, miR-185, Rab25

## Abstract

**Objective:**

*MicroRNAs (miRNAs)* expression has deregulated in several cancer types including breast cancer (BC). The present study aims at investigating the role, mechanism, clinical value of* let-7d *and *miR-185* in BC, and the possible correlation these miRNAs with Rab25.

**Materials and Methods:**

Tumor samples as well adjacent normal tissues (ANT) were acquired from fresh surgical specimens from 110 patients and the expression levels of* let-7d, miR-185, Rab25*, and* snail *were evaluated using real-time PCR. The immunohistochemical (IHC) process and western blot were done to detect the level of Rab25 and Snail protein expression in BC samples.

**Results:**

By comparing *miRNAs* expression profiles in clinical tissues of 110 patients using real-time PCR, *let-7d,* and *miR-185 *expression were dramatically downregulated in BC tissues (P < 0.05). Tumor size, stage, and lymph node metastasis were significantly related to *miRNAs* expression. Based on qRT-PCR and bioinformatics database analyses, we also recognized *Rab25* as a possible target of* miR-185* and *let-7d*.* Rab25* expression was enhanced in BC cells and associated inversely with the expression level of mentioned miRNAs. qRT-PCR, immunohistochemistry, and western blot studies verified that* Rab25* upregulation increased the levels of the snail, that key transcription factor of epithelial-mesenchymal transition (EMT).

**Conclusion:**

These findings demonstrated that* let-7d* and *miR-185 *inhibited EMT by targeting* Rab25* expression in BC. Therefore, targeting the *let-7d* and *miR-185/Rab25* interaction may offer new therapeutic opportunities for treating BC patients.

## Introduction

Breast cancer (BC) is the most prevalent malignancy among all women, and it is identified to be one of the deadliest cancers (Chatran et al., 2018; Jafari-Gharabaghlou et al., 2018; Rasouli et al., 2020). Despite the noticeable progress in the diagnosis of the early BC stage, most of the patients diagnosed with BC (~90%) die as a result of metastasis (Hosseini et al., 2016). Metastasis occurs through multi-step processes such as local invasion, migration, transport, and colonization (Montazeri et al., 2017; Mellatyar et al., 2018). One of the crucial factors for metastatic dissemination of cancer is a developmental process termed epithelial-mesenchymal transition (EMT) (Brabletz et al., 2018; Bahmanpour et al., 2019). EMT is a phenomenon in which cells lose their epithelial features and exhibit the migratory phenotype of mesenchymal cells (Aiello et al., 2018; Mittal, 2018). Snail, a master EMT-transcription factor, plays essential roles in multiple stages of embryonic development and is positively associated with tumor metastasis. Recent researches have highlighted that microRNAs (miRNAs) are important factors in EMT and cancer cell metastasis (Mohammadian et al., 2017a; Mohammadian et al., 2017b; Sheervalilou et al., 2017a). MiRNAs are short non-coding RNAs (containing ~22 nucleotides) that control target genes by sequence-specific binding with their 3′-untranslated regions (UTRs) (Mohammadian et al., 2016a; Mohammadian et al., 2016c; Sheervalilou et al., 2017b). Several reports have indicated that miRNAs interfere in several physiological and pathological processes such as cell growth, apoptosis, angiogenesis, and metastasis (Mohammadian et al., 2016b; Sheervalilou et al., 2016; Sheervalilou et al., 2019). Additionally, investigations have demonstrated the role of miRNAs in the metastatic cascade of BC. For example, miR-132 prevents proliferation and invasion of BC cells by targeting FOXA1 (Wei et al., 2018). MiR-200c suppresses metastasis of BC by decreasing Foxf2 (Zhang et al., 2017). MiR-19b-1 inhibits angiogenesis and migration by targeting VEZF1 in BC (Yin et al., 2018b). Thus, miRNAs and their targets can be considered a complex network wherein they can affect metastatic BC. According to studies, reduced expression of *let-7d* and *miR-185* has been detected in different types of cancers, such as colon, lung, gastric and ovarian cancer (García-Vázquez et al., 2018; Jiang et al., 2018). Let-7d belonging to the let-7 miRNA family has been recognized as a tumor suppressor miRNA, most likely by suppressing the expression of the high mobility group A2 (HMGA2) or k-RAS (Wang et al., 2016). Furthermore, miR-185 regulates several types of cellular signaling that are necessary for the biological properties of tumor cells (Chai et al., 2017). However, the molecular mechanisms of these miRNAs in regulating EMT in BC remain largely unknown. Many studies showed that these miRNAs regulated various genes, which one of the most well-known ones is the Ras family. Rab25 (Ras-related protein Rab-25) is known as a molecular switch that is implicated in various biological processes and signaling pathways under normal and cancerous conditions (Mitra et al., 2016). In the current study, Rab25 was found to be a target gene of both let-7d and miR-185. Over-expression of *Rab25 *can counteract the effect of let-7d and miR-185, restore BC malignant phenotypes, and mediate its efficacy in BC progression that can be associated with* Snail *expression. Our findings suggest that let-7d, miR-185, and Rab25 might be appropriate tools for intervention development to improve BC diagnosis and treatment.

## Materials and Methods

*Tissue samples*

Tumor samples as well adjacent normal tissues (ANT) were acquired from fresh surgical specimens from 110 patients who were treated at Noor-e-Nejat hospital, Tabriz, Iran from April 2016 to July 2018. The present research was ethically confirmed through the Ethics Committee of the Tabriz University of Medical Science (NO. IR.TBZMED.REC.1396.55). The clinicopathological data of these patients were collected with their signed written informed consent before inclusion in the current study. All fresh tissue specimens were gathered and instantly kept in liquid nitrogen at −80 °C. The histological studies were judged by two independent pathologists who were uninformed of the medical information of the corresponding patient.

*RT-PCR analysis*

Total cellular RNA was extracted from tissue samples according to the TRIzol reagent instructions (Invitrogen, USA). RNA was reverse-transcribed to complementary DNA (cDNA) by using QuantiTect cDNA synthesis kit (Qiagen, Germany) based on the manufacturers’ recommendations. RT-PCR with real-time quantitation was carried out by SYBR Green Master Mix and assessed on a Roche light cycler 96 (Germany) in triplicate. The sequence and details of the specific primers used to RT-PCR amplification of let-7d, miR-185, Rab25, snail, and GAPDH, as a reference gene, are listed in Table 1. PCR cycling conditions was as follow: 1 cycle at 95 ºC for 5 min, followed by 40 cycles of 95 ºC for 20 seconds and 72 °C for 30 seconds to 1 min. All samples were performed in triplicate and findings were computed by using the 2^−^^∆∆^^Ct^ method (Dadashpour et al., 2018). 

*Immunohistochemical staining*

The immunohistochemical (IHC) process was done to detect the level of Rab25 and Snail protein expression. Tumor tissues were first fixed in 10% formalin-PBS buffer, embedded in paraffin, sectioned (4 μm thickness), and was mounted on glass slides. Then, diluted hydrogen peroxide (3%) was utilized to hinder the endogenous peroxidase activity. All the retrievals were performed in citrate buffer (pH 6·0) using microwave after that 1 h incubation with a mouse anti-Rab25 monoclonal antibody (1: 250, Abcam Inc, MA, USA) and rabbit anti-snail monoclonal antibody (1:100, Santa Cruz, Santa Cruz, CA, USA) overnight at 4°C. Negative controls were also obtained by the omission of specific primary antibodies. Then, tissue specimens were incubated with anti-mouse and the anti-rabbit secondary antibody, conjugated to HRP, at ambient temperature for 30 min. 3,3′−diaminobenzidine (DAB) tetrahydrochloride substrate was utilized for about 5 min to color development. Samples were counterstained with 0.1% hematoxylin and, finally, IHC stained slide investigated by a light microscope (Pilehvar-Soltanahmadi et al., 2017). 

*Immunohistochemical analysis*

Assessment of IHC data was conducted by two different researchers who were blinded about clinical data of patients. The IHC results were stratified into 4 levels: 0, no staining; 1, weak; 2, moderate and 3, strong. The percent of the positive staining cells were scored as follows: 1 for <25%, 2 for 25-50%, 3 for 51–75%, and 4 for >75%. Final results were presented as follows: - (0 score, absent), + (1–4 score, weakly positive), ++ (5-8 score, moderately positive) and +++ (9-12 score, strongly positive). The scores of <5 was regarded as negative and the scores of ≥5 were defined as positive.

*Western blotting*

We performed western blot to identify Rab25 and Snail protein expression in BC samples. The protein content of tissue lysates was separated using 10% SDS-polyacrylamide gels and moved to polyvinyl difluoride membranes (Roche Diagnostics GmbH). Once being blocked with 5% defatted dry milk, the membranes were incubated with the primary antibodies against Rab25, Snail, and GAPDH at 4˚C overnight. Afterward, the immunoreactive proteins were incubated with HRP-conjugated secondary antibodies (1:3,000, Razi Biotech Co, Tehran, Iran) at ambient temperature for 2 h and then visualized by using ECL detection Kit (Bio-Rad). The intensity of each western blot band was counted via ImageJ, version 1.44 software (National Institutes of Health, Bethesda, USA) and normalized to the respective GAPDH loading control (Sadeghi-Soureh et al., 2020).

*Statistical analysis*

The statistical data analysis was conducted applying the SPSS 16.0 (Chicago, IL, USA) or Graph Pad Prism 6 (San Diego, CA, USA). Comparisons between the difference in means values among mRNA levels of BC and ANT samples were determined using the Mann–Whitney U test. One-way analysis of variance (ANOVA) was applied to assess the statistical differences in *mRNA* expression levels between the different histologic tumor grades. The statistical relationship among mRNA or protein levels of each group and clinicopathological features were analyzed using a Chi-Square, Student’s T-test or Mann-Whitney test. p-value <0.05 was considered to show a statistically significant difference.

## Results

*Let-7d and miR-185 expression was decreased in BC samples and was associated with clinicopathological criteria*

**Figure 1:**
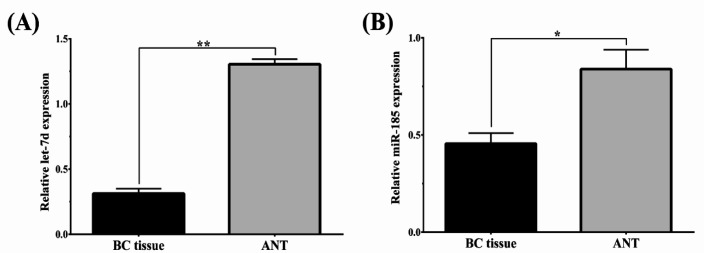
Expression of* let-7d* and *miR-185* was Downregulated in Breast Cancer (BC) Tissues. Quantitative PCR of let-7d and miR-185 in BC and adjacent normal specimens (ANT) (n=110). All samples were repeated 3 times and data were normalized to GAPDH. * p < 0.05, ** p < 0.01

We investigated the potential effect of let-7d and miR-185 in BC development. We first examined the relative level of *let-7d* and *miR-185* expression in 110 specimens of BC and ANT tissues. Let-7d and miR-185 were suppressed 4 and 1.8-fold, respectively, in BC compared to ANT (both P < 0.05) (Figures 1A and B). Table 2 summarizes the correlation of let-7d and miR-185 with the clinicopathological criteria of the patients reviewed. In brief, no statistically significant difference was observed between *let-7d* and *miR-185* and age, histological grade, expression of estrogen receptor (ER), progesterone receptor (PR), and HER-2. On the contrary, the results demonstrated that let-7d and miR-185 were significantly associated with tumor size, tumor stage, and lymph node metastasis. Our results indicated that downregulation of *let-7d* and *miR-185* expression in BC tissues might cause metastasis.

*Rab25 and Snail expression was increased in BC samples and was associated with clinicopathological criteria*

**Figure 2:**
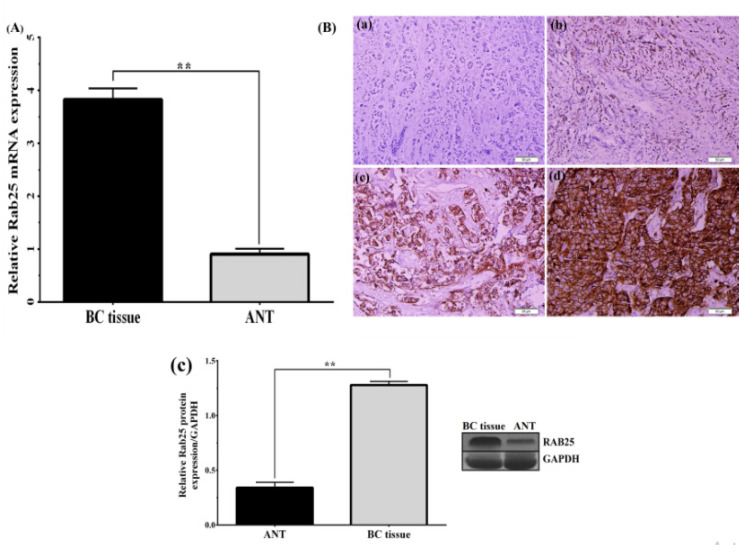
Expression of *Rab25* was up-Regulated in Breast Cancer (BC) Ttissues. (A) Immunohistochemical staining of Rab25 expression in BC tissues. (a) 0 (no staining); (b) 1+ (weak); (c) +2 (moderate); and (d) +3 (strong). (B) Relative expression of Rab25 was detected by using the quantitative PCR in breast cancer and paired adjacent normal tissues (ANT), **p < 0.01. (C) Rab25 protein expressions was determined by using the western blot assay. * p < 0.05

**Figure 3:**
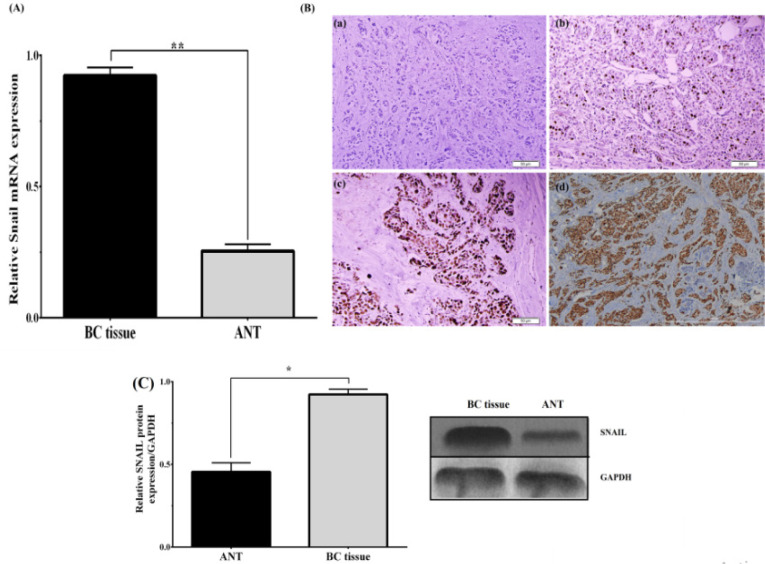
Expression of *Snail *was up-Regulated in Breast Cancer (BC) Tissues. (A) Immunohistochemical staining of the Snail expression in BC tissues. (a) 0 (no staining); (b) 1+ (weak); (c) +2 (moderate); and (d) +3 (strong). (B) Relative expression of Snail was detected by using the quantitative PCR in BC and paired adjacent normal tissues (ANT), ** p < 0.01. (C) Snail protein expressions was determined by using the western blot assay. * p < 0.05

The protein levels of Rab25 and Snail were detected by the IHC staining intensity in both BC and paired ANT tissues. In this case, *Rab25* was expressed primarily in the cytoplasm, along with minimal expression in the nuclei of BC cells (Figure 2A). On the contrary, Snail staining was localized predominantly in the nucleus, and no positive staining was detected in the cytoplasm of BC cells. (Figure 3A). The positive expression rates of *Rab25* and *Snail *expression in BC tissues were 82.7% (91/110) and 85.4% (94/110), respectively, is markedly higher than those of the relevant ANT (19%,21/110) and (35.4%, 39/110). To confirm IHC results, we also conducted quantitative-PCR to examine the *Rab25 s* and *Snail mRNA* expression level in all BC and ANT cases. The level of Rab25 and Snail mRNA was higher in the BC group than in the ANT group (both P =0.001) (Figures 2Band 3B). Table 3 summarizes the associations of Rab25 and Snail protein expressions with various clinicopathological criteria. Our results indicated that *Rab25* overexpression was associated with the tumor size (P = 0.021), lymph-node metastasis (P = 0.032), histological grade (P = 0.047) and pathological stage (P = 0.038) of BC. Besides, we detected a positive association between the *Snail* expression level and the tumor size (p=0.036), pathological stage (P =0.049), and lymph node metastasis (P =0.018). It is worth noting that similar results were found when the relationship was investigated between *mRNA* expression levels and clinicopathological parameters. Table 2 shows the data. The results showed that the *Rab25* and* Snail *expression in BC tissues was significantly higher than that in ANT tissues. To confirm the results of IHC and qPCR analyses and to further investigate the effect of Rab25 in induced EMT, we determined the expression levels of *Rab25* and* Snail* by western blotting. Interestingly, our findings confirmed that the upregulation expression of *Rab25* increased Snail to a certain extent (Figures 2C and 3C). As a result, these results strongly indicate that Rab25 promotes the EMT phenotype in BC cells through upregulation expression of *Snail*.

*Rab25 expression is positively correlated with the Snail expression in BC tissues*

The Spearman’s rank correlation coefficient was used to examine the relationship between Rab25 and snail protein expression levels. As Table 4 shows, Rab25 protein expression is positively correlated with the expression level of *Snail*. Out of sixty-seven Rab25 positive (++/+++) cases, fifty-two (77.6%) BC patients positively expressed Snail (++/+++), while the co-expression of *Rab25* and *Snail* was detected in two (25%) of the eight Rab25 positive cases in ANT tissues. Moreover, from sixteen BC samples with negative staining (−/+) for Snail, fourteen (87.5%) patients indicated IHC negative (−/+) for Rab25. Our finding revealed that the *Rab25* and *Snail* expression levels were significantly associated with each other in all BC (r = 0.513, p =0.011) and ANT samples (r= 0.263, p = 0.046).

*Correlation between let-7d and miR-185 and Rab25 in BC tissues*

**Figure 4:**
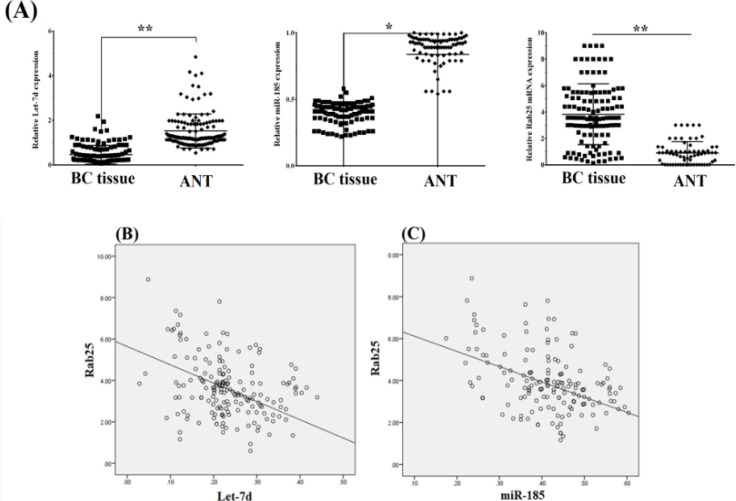
*Rab25* was the Direct Target of Both *let-7d *and *miR-185*. (A) Relative expression of *let-7d *and *miR-185 *compared to *Rab25* expression in breast cancer (BC) tissues. (B) Scatter plot displaying the Spearman's correlation between the *let-7d* and *Rab25* expression in BC and adjacent normal tissues (ANT). (C) Scatter plot displaying the Spearman's correlation between *miR-185* and *Rab25* expression in BC and ANT samples

Recent studies have reported that the reduction of some miRNAs was found to be related to higher levels of expression of Rab proteins in different cancers (Shahabi et al., 2019; Yan et al., 2019). First, to ascertain the miRNAs targeting Rab25, we excavated target genes through two bioinformatics databases (miRDB and Target Scan) and provided a list of miRNAs targeting Rab25. Afterward, data mining techniques were performed, and miRNAs that significantly implicated the development in BC progression and metastasis phenotype were selected. According to the findings, we found that Rab25 might be one of the target genes of *let-7d *and *miRNA-185*. Moreover, the qPCR analysis showed that diminished expression of these miRNAs significantly downregulated the *Rab25* expression in BC tissues rather than in ANT (Figures 4Aand 4B). Correlation analysis of *let-7d, miR-185*, and *Rab25* expression in BC tissues demonstrated an inverse relationship between declared miRNAs and Rab25 (Figure 4C). Overall, these results suggest that Rab25 is downregulated by let-7d and miRNA-185.

**Table 1:** Sequences of l*et-7d, miR-185, Rab25*, *Snail* and *GAPDH* Primers in Real-Time PCR

Gene	Primer sequences
*Let-7d*	Forward: 5′-GCGAACTGTTTGCAGAGG-3′
	Reverse: 5′-CAGTGCGTGTCGTGGAGT-3′
*miR-185*	Forward: 5′-TGCGGGTGCTCGCTTCGGCAGC-3′
	Reverse: 5′-CCAGTGCAGGGTCCGAGGT-3′
*Rab25*	Forward: 5′-ATCTTCTCCTCGCTTCTGG-3′
	Reverse: 5′-GCCTGCTGGCTGGTTATCA-3′
*Snail*	Forward: 5′-CAATGCTCATCTGGGACTCT-3′
	Reverse: 5′-TTTCCCACTGTCCTCATCTG-3′
*GAPDH*	Forward: 5′-TGTGGGCATCAATGGATTTGG-3′
	Reverse: 5′-ACACCATGTATTCCGGGTCAAT-3′

**Table 2:** Association between Clinicopathological Characteristics and mRNA Expression of* let-7d, miR-185, Rab25 *and *Snail *in 110 Patients with Breast Cancer

Clinical pathological criteria	*RAB25*			*Snail*		*Let-7d*		*miR-185*	
	No.	expression (Mean ± SD)	P	expression (Mean ± SD)	P	expression (Mean ± SD)	P	expression (Mean ± SD)	P
Age			0.569		0.807		0.31		0.563
<50	75	3.88 ± 1.57		0.95 ± 0.30		0.19 ± 0.06		0.32 ± 0.19	
>50	35	4.07 ± 1.74		0.93 ± 0.38		0.17 ± 0.07		0.30 ± 0.17	
histological grade			0.047		0.059		0.11		0.063
I	21	3.46 ± 1.51		1.01 ± 0.56		0.22 ± .08		0.43 ± 0.07	.
II	79	3.89 ± 1.56		1.21 ± 0.75		0.18 ± .07		0.38 ± 0.08	
III	10	4.99 ± 1.62		1.32 ± 0.82		0.16 ± .08		0.239 ± 0.16	
PR			0.968		0.894		0.319		0.883
Negative	34	3.97 ± 2.38		0.96 ± 0.33		0.17 ± 0.07		0.39 ± 0.83	
Positive	76	3.94 ± 1.54		0.94 ± 0.31		0.19 ± 0.06		0.40 ± 0.84	
ER			0.475		0.33		0.902		0.961
Negative	27	4.02 ± 1.73		0.84 ± 0.16		0.19 ± 0.10		0.40 ± 0.23	
Positive	83	3.93 ± 1.62		0.95 ± 0.33		0.18 ± 0.06		0.38 ± 0.38	
HER2			0.591		0.104		0.797		0.928
Negative	99	3.96 ± 1.63		0.95 ± 0.33		0.19 ± 0.01		0.39 ± 0.08	
Positive	11	3.59 ± 1.36		0.73 ± 0.15		0.18 ± 0.03		0.39 ± 0.09	
Lymph node metastasis			0.032		0.018		0.011		0.041
Negative	46	3.51 ± 1.61		0.93 ± 0.32		0.21 ± 0.11		0.41 ± 0.15	
Positive	64	4.22 ± 1.57		1.03 ± 0.33		0.17 ± 0.08		0.32 ± 0.18	
Stage			0.038		0.049		0.043		0.047
1	16	3.48 ± 0.585		0.967 ± 0.18		0.21 ± 0.15		0.43 ± 0.15	
2	72	3.69 ± 1.40		0.894 ± 0.30		0.19 ± 0.07		0.40 ± 0.08	
3	18	3.88 ± 1.69		1.14 ± 0.38		0.16 ± 0.08		0.35 ± 0.11	
4	4	4.18 ± 1.62		0.902 ± 0.07		0.15 ± 0.07		0.35 ± 0.12	
Tumor size			0.021		0.036		0.009		0.049
<20mm	39	3.54 ± 1.51		0.89 ± 0.27		0.21 ± 0.05		0.42 ± 0.07	
20-49mm	61	3.95 ± 1.50		0.93 ± 0.32		0.18 ± 0.06		0.39 ± 0.08	
>50mm	10	4.94 ± 1.94		1.16 ± 0.48		0.15 ± 0.05		0.34 ± 0.11	

**Table 3:** Correlation between Clinicopathological Characteristics and Protein Expression of *Rab25* and *Snail *in 110 Patients with Breast Cancer

Clinical pathological criteria			*RAB25*			*Snail*		
		No.	Negative	Positive	P	Negative	Positive	P
Age					0.136			0.384
	<50	75	15	60		9	66	
	>50	35	9	26		7	28	
histological grade					0.041			0.038
	I	21	7	14		4	17	
	II	79	17	62		12	67	
	III	10	1	9		1	9	
PR					0.416			0.208
	Negative	34	13	21		16	18	
	Positive	76	22	54		11	51	
ER					0.217			0.195
	Negative	27	9	18		8	19	
	Positive	83	16	67		22	61	
HER2					0.753			0.881
	Negative	99	23	76		21	78	
	Positive	11	3	8		2	9	
Lymph node metastasis					0.009			0.001
	Negative	46	20	26		13	33	
	Positive	64	4	60		3	61	
Stage					0.015			0.046
	1	16	7	9		4	12	
	2	72	17	55		11	61	
	3	18	2	16		1	17	
	4	4	1	3		1	3	
Tumor size					0.032			0.146
	<20mm	39	11	28		9	31	
	20-49mm	61	12	49		5	55	
	>50mm	10	2	8		2	8	

**Table 4:** Association between *Rab25* and *Snail* Expression in Breast Cancer

		NO.	*RAB25* expression				Correlation coefficient
			-	+	++	+++	
*Snail* expression	BC tissues	-	11	3	1	1	r= 0.513 (p = 0.011)
		+	5	12	20	5	
		++	3	8	17	16	
		+++	0	1	3	4	
			-	+	++	+++	
*Snail* expression	ANT	-	65	5	0	1	r= 0.263 (p= 0.046)
		+	15	10	5	0	
		++	4	1	1	1	
		+++	0	2	0	0	

## Discussion

miRNAs play vital roles in regulating the expression of cancer-related genes expression and are implicated in cancer development and progression (Kafshdooz et al., 2018; Tiwari et al., 2018; Norouzi et al., 2019; Sheervalilou et al., 2020). Previous studies have indicated revealed that the let-7d level is strongly down-regulated downregulated in various cancer cells. Let-7d directly targets c-Myc, HMGA2, CCL7, PBX3 and COL3A1 (Ramberg et al., 2011; Su et al., 2014; Ye et al., 2014), and so inhibits thereby inhibiting cancer cell invasion and metastasis. Another reports report found that miR185 was able to regulate cellular proliferation, as well as DNA-damage response and repair (Wang et al., 2013; He et al., 2016). The relative expression of* let-7d* and *miR-185* are lower in a variety of various cancer tissues. Besides, low expression of the mentioned miRNAs is related to large tumor size and advanced metastatic cancer (Wei et al., 2018). Consistent with previous reports (Fu et al., 2014; Wei et al., 2018), we discovered that *let-7d* and *miR-185* expression was markedly reduced in BC tissues versus than in their paired ANTs, and proven both serve served as a tumor suppressor gene in BC. EMT is considered an important molecular event in tumor invasion and migration (Jolly et al., 2017). It is now well known that miRNAs can target the EMT regulatory factors to promote or inhibit EMT in BC cells (Zhao et al., 2017). For instance, miR-497 suppresses EMT and metastasis of BC cells by targeting SMAD7 (Liu et al., 2016). MiR-125b negatively regulates EMT and influence influences BC invasion and migration by targeting MAP2K7 (Hong et al., 2016). Other miRNAs, such as miR-206, miR-381, miR-520c-3p, miR-143, and miR-153 inhibit the EMT-related BC and exhibit anticancer properties via several target genes and signaling pathways (Li et al., 2015; Zhai et al., 2016; Tang et al., 2017; Xue et al., 2017). Our results prove that Let-7d and miR-185 reverse EMT in BC cells. Rab25 has been implicated in diverse diseases, many of which are linked to the hijacking and use of intracellular trafficking pathways though through types of pathogens (Cheng et al., 2004). Additionally, overexpression of *Rab25* increased the tumorigenicity and invasiveness in BC cancer (Wang et al., 2015). Our findings indicate that Rab25 stimulates the EMT-related cell invasion of BC. In accordance with our findings, Yin et al., (2018a) also exhibited unraveled that downregulation of the level of Rab25 in BC cells substantially suppressed suppresses the invasive motility and is related to the induction of vimentin and reduction of E-cadherin, which are the key markers of EMT). It is well known that Obviously, miRNAs could negatively regulate target genes via binding to 3′-UTR mRNAs, and *let-7d* and *miR-185 *downregulated downregulate the *Rab25* expression. In conclusion, this study implied demonstrated that let-7d and miR-185 downregulation was associated with markedly significantly increased *Rab25* expression, thereby reinforces reinforcing the BC invasion and metastasis via the snail-driven EMT. These results describe the mechanism underlying the tumor suppressor functions of let-7d and miR-185 and demonstrate that the signal pathway let-7d and miR-185/Rab25 might be a potential target for BC treatment.

In conclusion, this study demonstrated that *let-7d* and *miR-185* downregulation was associated with significantly increased *Rab2*5 expression, thereby reinforcing the BC invasion and metastasis via the snail-driven EMT. These results describe the mechanism underlying the tumor suppressor functions of let-7d and miR-185 and demonstrate that the signal pathway let-7d and miR-185/Rab25 may be a potential target for BC treatment.
